# Inflammatory Biomarkers to Predict Major Adverse Cardiovascular Events in Patients with Carotid Artery Stenosis

**DOI:** 10.3390/medicina60060997

**Published:** 2024-06-18

**Authors:** Ben Li, Farah Shaikh, Abdelrahman Zamzam, Rawand Abdin, Mohammad Qadura

**Affiliations:** 1 Department of Surgery, University of Toronto, Toronto, ON M5S 1A1, Canada; benx.li@mail.utoronto.ca; 2 Division of Vascular Surgery, St. Michael’s Hospital, University of Toronto, Toronto, ON M5S 1A1, Canada; farah.shaikh@unityhealth.to (F.S.); abdelrahman.zamzam@gmail.com (A.Z.); 3 Institute of Medical Science, University of Toronto, Toronto, ON M5S 1A1, Canada; 4 Temerty Centre for Artificial Intelligence Research and Education in Medicine, University of Toronto, Toronto, ON M5S 1A1, Canada; 5 Department of Medicine, McMaster University, Hamilton, ON L8S 4L8, Canada; rawand.abdin@medportal.ca; 6 Li Ka Shing Knowledge Institute, St. Michael’s Hospital, University of Toronto, Toronto, ON M5S 1A1, Canada

**Keywords:** inflammatory proteins, IL-6, CD163, prognosis, carotid artery stenosis

## Abstract

*Background and Objectives:* Inflammatory proteins and their prognostic value in patients with carotid artery stenosis (CAS) have not been adequately studied. Herein, we identified CAS-specific biomarkers from a large pool of inflammatory proteins and assessed the ability of these biomarkers to predict adverse events in individuals with CAS. *Materials and Methods:* Samples of blood were prospectively obtained from 336 individuals (290 with CAS and 46 without CAS). Plasma concentrations of 29 inflammatory proteins were determined at recruitment, and the patients were followed for 24 months. The outcome of interest was a major adverse cardiovascular event (MACE; composite of stroke, myocardial infarction, or death). The differences in plasma protein concentrations between patients with vs. without a 2-year MACE were determined using the independent *t*-test or Mann–Whitney *U* test to identify CAS-specific prognostic biomarkers. Kaplan–Meier and Cox proportional hazards analyses with adjustment for baseline demographic and clinical characteristics were performed to assess the prognostic value of differentially expressed inflammatory proteins in predicting a 2-year MACE in patients with CAS. *Results:* The mean age of the cohort was 68.8 (SD 10.2) years and 39% were female. The plasma concentrations of two inflammatory proteins were significantly higher in individuals with a 2-year MACE relative to those without a 2-year MACE: IL-6 (5.07 (SD 4.66) vs. 3.36 (SD 4.04) pg/mL, *p* = 0.03) and CD163 (233.825 (SD 230.306) vs. 159.673 (SD 175.669) pg/mL, *p* = 0.033). Over a follow-up period of 2 years, individuals with elevated levels of IL-6 were more likely to develop MACE (HR 1.269 (95% CI 1.122–1.639), *p* = 0.042). Similarly, over a 2-year period, patients with high levels of CD163 were more likely to develop MACE (HR 1.413 (95% CI 1.022–1.954), *p* = 0.036). *Conclusions:* The plasma levels of inflammatory proteins IL-6 and CD163 are independently associated with adverse outcomes in individuals with CAS. These CAS-specific prognostic biomarkers may assist in the risk stratification of patients at an elevated risk of a MACE and subsequently guide further vascular evaluation, specialist referrals, and aggressive medical/surgical management, thereby improving outcomes for patients with CAS.

## 1. Introduction

Carotid artery stenosis (CAS) involves atherosclerosis in the carotid arteries and is a major contributor to ischemic strokes, which causes important morbidity/mortality globally as highlighted in recent studies by Qaja et al. (2022) and Donkor and colleagues (2018) [1,2]. However, the condition is heterogeneous in that some patients will remain asymptomatic throughout their disease course, while others will suffer debilitating strokes leading to permanent disability or death [1,3]. Currently, it is challenging to predict the clinical course of patients with CAS [4]. Although imaging to determine the severity of atherosclerosis may help predict adverse events, it comes with limitations [5,6]. For example, some patients with severe stenosis may remain asymptomatic while others with moderate stenosis may develop stroke [5,6]. One potential reason is that imaging alone does not fully consider the molecular mechanisms by which atherosclerotic plaques lead to strokes [7]. Therefore, identifying biomarkers associated with adverse outcomes in patients with CAS may provide important information to support the prognosis of patients with CAS to guide further personalized evaluation and treatment [7].

Inflammatory proteins have been demonstrated to be involved in cardiovascular disease development, including the cluster of differentiation 163 (CD163) [8] and interleukin-6 (IL-6) [9]. The mechanisms by which IL-6 and CD163 may increase the risk of major adverse cardiovascular events in patients with carotid artery stenosis include the facilitation of pro-inflammatory and pro-atherogenic pathways that may contribute to plaque progression and instability leading to adverse cardiovascular outcomes [10,11,12,13]. Over 20 inflammatory cardiovascular disease biomarkers have been investigated [14,15,16,17,18,19,20,21,22,23]. We selected 29 specific inflammatory biomarkers for analysis because they have been thoroughly studied and show robust associations with cardiovascular diseases with potential applications to CAS [14,15,16,17,18,19,20,21,22,23]. Although existing papers have shown correlations between these proteins and cardiovascular diseases, few have assessed their implications for prognosis in CAS by assessing their ability to predict adverse events independently of other clinical characteristics [14,15,16,17,18,19,20,21,22,23]. The reason this study focused specifically on CD136 and IL-6 is that these proteins are involved in various metabolic pathways that promote inflammation and atherogenesis, which are fundamental contributors to CAS development and progression [8,9]. Therefore, CD136 and IL-6 may have strong prognostic value for CAS-related adverse events [8,9]. The novelty of this study stems from the fact that we identified CAS-specific prognostic biomarkers from a large panel of circulating proteins and assessed their ability to independently predict CAS-related outcomes using statistical tests that adjusted for baseline characteristics, which has not been performed by previous studies [14,15,16,17,18,19,20,21,22,23]. This study primarily focuses on identifying CAS-specific prognostic biomarkers from a large pool of inflammatory proteins and characterizing the ability of these biomarkers to predict long-term adverse cardiovascular events in individuals with CAS. The findings of this study have the potential to improve outcomes for patients with CAS by identifying high-risk patients who may benefit from further vascular evaluation and aggressive medical/surgical management.

## 2. Materials and Methods

### 2.1. Ethics Approval

This project was granted approval by the Unity Health Toronto research ethics board on 8 February 2017 (REB # 16-365). All participants provided informed consent, and all procedures were conducted according to the principles outlined in the Declaration of Helsinki [24].

### 2.2. Study Design

This prognostic study was conducted and reported using the transparent reporting of a multivariable prediction model for individual prognosis or diagnosis (TRIPOD) statement [25]. Specifically, this study was designed to identify CAS-specific prognostic biomarkers from a large panel of circulating proteins using a prospectively recruited cohort and evaluate the ability of these proteins to independently predict CAS-related adverse events.

### 2.3. Patient Recruitment

This study prospectively recruited patients with and without CAS who presented to ambulatory clinics at our institution between November 2019 and January 2022. CAS was defined as >50% stenosis of at least 1 of the carotid arteries on a duplex ultrasound using the North American Symptomatic Carotid Endarterectomy Trial (NASCET) criteria [26]. The duplex ultrasound study was performed at the time of patient recruitment, and the reason for a patient receiving a duplex ultrasound study was for suspected carotid artery stenosis based on neurological symptoms or cardiovascular risk factors as determined based on clinical assessment by a vascular surgeon. Non-CAS was defined as <50% stenosis of both carotid arteries on a duplex ultrasound based on the NASCET criteria [26] and included patients with non-CAS pathologies including peripheral artery disease, abdominal aortic aneurysm, and varicose veins. Patients received standard CAS guideline-directed cardiovascular risk reduction therapy including acetylsalicylic acid and statins; however, they did not receive any biological or anti-inflammatory medications that are known to influence the modulation of inflammatory proteins [27,28]. Patients with acute coronary syndrome, elevated troponin, or inflammatory disorders requiring biological anti-inflammatory medications within the previous 3 months were excluded.

### 2.4. Baseline Characteristics

The baseline characteristics documented in this study included age, sex, hypertension (diastolic blood pressure ≥ 80 mmHg, systolic blood pressure ≥ 130 mmHg, or taking blood pressure-lowering therapy [29,30]), dyslipidemia (triglyceride > 1.7 mmol/L, total cholesterol > 5.2 mmol/L, or taking lipid-lowering therapy [29,30]), diabetes (hemoglobin A1c ≥ 6.5% or taking an antidiabetic medication [29,30]), current smoking habits, congestive heart failure (CHF), coronary artery disease (CAD), and history of stroke. Cardiovascular risk factors were defined based on the American College of Cardiology guidelines [29,30].

### 2.5. Quantification of Plasma Inflammatory Biomarker Levels

Samples of blood were obtained from the participants, and plasma concentrations of 29 inflammatory proteins were assessed in duplicate using the LUMINEX assay (Bio-Techne, Minneapolis, MN, USA) [31]. The following proteins were selected for analysis because of their involvement in metabolic processes that are associated with atherosclerosis and important associations with cardiovascular diseases: chemokine (C-C motif) ligand 1 (CCL1)/TCA-3, tumor necrosis factor alpha (TNF-α), MMP-8, CD163, bone morphogenetic protein 10 (BMP-10), BMP-7, BMP-4, insulin-like growth factor-binding protein-1 (IGFBP-1), CCL3/macrophage inflammatory protein-1 alpha (MIP-1a), CCL13/MIP-1 delta, CCL4/MIP-1b, chemokine (C-X-C motif) ligand 16 (CXCL16), oxteoactivin/glycoprotein (transmembrane) NMB (GPNMB) resistin, CXCL9/monokine induced by gamma (MIG), regenerating family member 3 alpha (Reg3α), interferon gamma (IFNy), proganulin (PGRN), CCL17/thymus and activation regulated chemokine (TARC), HTRA2/Omi, Serpin A12, Serpin B3/squamous cell carcinoma antigen 1 (SCCA1), carcinoembryonic antigen-related cell adhesion molecule 1 (CEACAM1/CD66a), CCL11/Eotaxin, IL-6, MMP-7, MMP-10, CCL-2/MCP-1, and TFPI. By analyzing a large number of inflammatory proteins, we aimed to identify novel CAS-specific biomarkers. Specifically, by narrowing down a large pool of inflammatory proteins to a few proteins that have strong predictive ability for CAS-related adverse events, we can identify highly accurate prognostic biomarkers for CAS. Prior to analyzing the samples, bead kits from Fluidics Verification and Calibration (Luminex Corp, Austin, TX, USA) [32] were utilized for the calibration of the MagPix analyzer (Luminex Corp, Austin, TX, USA) [33]. To prevent any intra- and inter-assay variability, all analyses were performed in 1 day. Sample inter- and intra-assay coefficients of variability were below 10%. A minimum of fifty beads for each protein were analyzed using Luminex xPonent version 4.3 [34].

### 2.6. Follow-Up and Outcomes

Follow-up visits were conducted at 12 and 24 months after the baseline assessment. At each visit, a complete evaluation was performed including a repeat carotid duplex ultrasound and an assessment of changes in clinical status and study outcomes. The outcome of interest was a 2-year major adverse cardiovascular event (MACE; composite of stroke, myocardial infarction, or death). Myocardial infarction was defined as acute myocardial ischemia based on clinical symptoms, electrocardiogram changes, and troponin elevation. Stroke was defined as ipsilateral or contralateral neurological deficits persisting for >24 h. Death was defined as all-cause mortality.

### 2.7. Statistical Analysis

The baseline characteristics of our cohort were reported as means (standard deviations) or numbers (proportions). The differences in baseline characteristics between patients with vs. without CAS were assessed using the independent *t*-test for continuous variables (age) and chi-square test for categorical variables (sex, hypertension, dyslipidemia, diabetes, current smoking, CAD, CHF, and previous stroke). Plasma protein levels were compared between CAS patients with and without a 2-year MACE using the independent *t*-test if normally distributed or the Mann–Whitney *U* test if non-normally distributed. Proteins that were expressed differentially in individuals with and without a 2-year MACE were used in further analyses for model development. Adjusted hazard ratios (HR) for a 2-year MACE per one unit increase in each differentially expressed inflammatory biomarker (IL-6 and CD163) were assessed using Cox proportional hazards analysis, controlling for age, sex, hypertension, dyslipidemia, diabetes, current smoking, CAD, CHF, and previous stroke. Given that the baseline condition may be poorer in patients with CAS compared to patients without CAS, this adjusted analysis allowed for the controlling of baseline differences to assess the independent association between protein biomarkers and a 2-year MACE. Freedom from a MACE over 2 years in individuals with low vs. high plasma levels of IL-6 and CD163 was analyzed with Kaplan–Meier curves and compared using Cox proportional hazards analysis. Low and high plasma levels of IL-6 and CD163 were determined based on the median plasma concentrations of these proteins in our cohort. This stratified analysis allowed us to understand the clinical significance of elevated levels of these plasma proteins. Specifically, it helps clinicians understand the trajectory of a patient with low vs. high protein levels over a 2-year period in terms of MACE risk. Patients lost to follow-up were censored. The sample size of this study was 336 patients (290 with CAS and 46 without CAS), which was determined using a validated sample size calculator for clinical prediction models [35]. A larger number of patients with CAS were recruited to ensure that an adequate number of patients with the disease of interest were enrolled to provide an adequate sample size to identify CAS-specific prognostic biomarkers. Statistical significance was determined at 2-tailed *p* below 0.05. SPSS software version 23 was used to perform all statistical analyses [36].

## 3. Results

### 3.1. Patients

Overall, 336 patients were included (290 with CAS and 46 without CAS). Patients with CAS were older (mean age 71.60 (SD 8.55) vs. 63.76 (SD 13.38) years, *p* < 0.001) and a greater proportion had dyslipidemia (82% vs. 51%, *p* < 0.001), hypertension (78% vs. 55%, *p* < 0.001), diabetes (31% vs. 22%, *p* < 0.03), CAD (37% vs. 27%, *p* < 0.03), and a prior stroke (34% vs. 22%, *p* = 0.005) (Table 1). A complete follow-up was obtained for 46 (100%) patients without CAS at 1 year and 2 years and for 258 (89.0%) and 239 (82.4%) patients with CAS at 1 year and 2 years, respectively.

### 3.2. Circulating Inflammatory Protein Levels

From an initial panel of 29 inflammatory proteins, we identified two proteins that were significantly elevated in patients with CAS who developed a 2-year MACE compared to those who did not develop a 2-year MACE: IL-6 (5.07 (SD 4.66) vs. 3.36 (SD 4.04) pg/mL, *p* = 0.03) and CD163 (233.825 (SD 230.306) vs. 159.673 (SD 175.669) pg/mL, *p* = 0.033) (Table 2). Given that the goal of this study was to build a prognostic model for a 2-year MACE in patients with CAS, protein levels were compared in patients with CAS who developed vs. did not develop a 2-year MACE to identify proteins that were associated with a 2-year MACE in patients with CAS. These two proteins (IL-6 and CD163) were therefore analyzed further.

### 3.3. Associations between Inflammatory Biomarkers (IL-6 and CD163) and MACE

At 1 year of follow-up, 23 (7.93%) patients developed a MACE in the CAS cohort, while zero patients developed a MACE in the non-CAS cohort. At 2 years of follow-up, 30 (10.34%) patients developed MACE in the CAS cohort, while four (8.70%) patients developed MACE in the non-CAS cohort (Table 3). There were significant correlations between every one unit increase in the two inflammatory proteins and a 2-year MACE: IL-6 (adjusted HR 1.269 (95% CI 1.122–1.639), *p* = 0.042) and CD163 (adjusted HR 1.413 (95% CI 1.022–1.954), *p* = 0.036) (Table 4).

### 3.4. Kaplan–Meier and Cox Proportional Hazards Analysis

**Figure 1:**
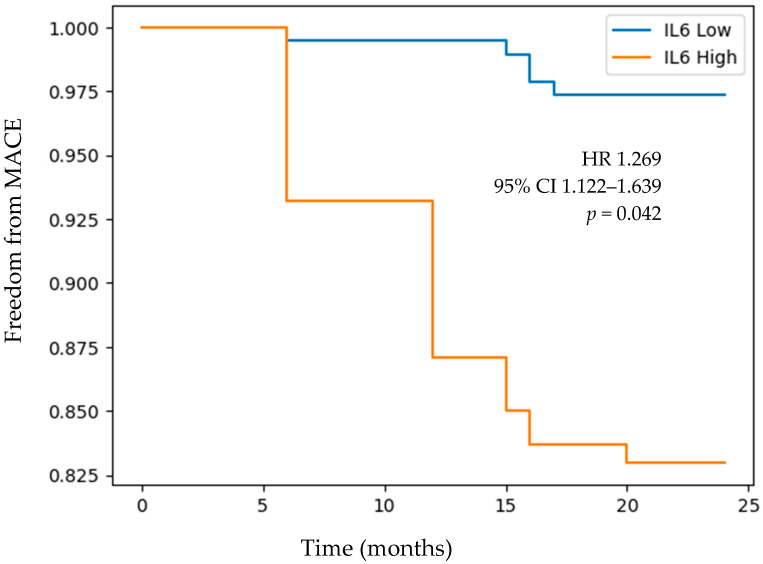
Kaplan–Meier curve of freedom from a MACE over 2 years of follow-up in individuals with low vs. high IL-6 levels. Stratification of patients into low and high IL-6 groups was determined based on the median plasma concentration of IL-6 in our patient sample. Abbreviations: IL-6 (interleukin-6) and MACE (major adverse cardiovascular event; composite of stroke, myocardial infarction, or death).

**Figure 2:**
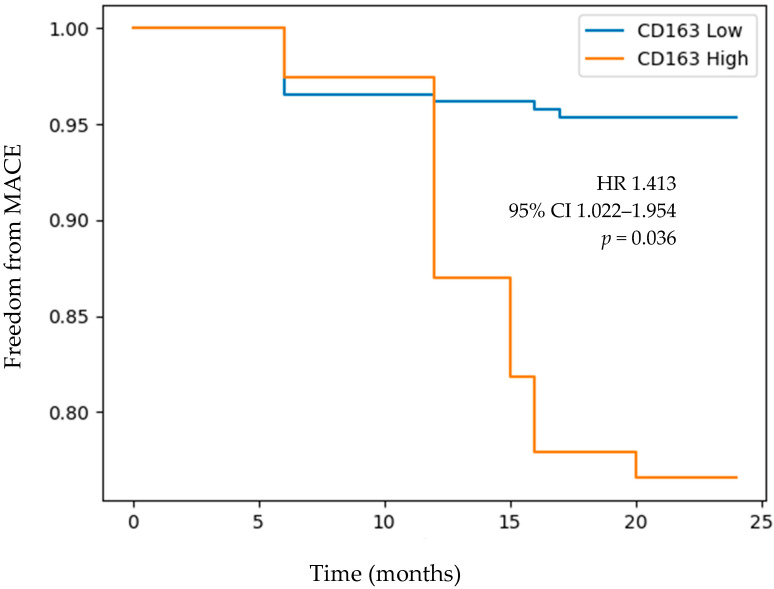
Kaplan–Meier curve of freedom from a MACE over 2 years of follow-up in individuals with low vs. high CD163 levels. Stratification of patients into low and high CD163 groups was determined based on the median plasma concentration of CD163 in our patient sample. Abbreviations: CD163 (cluster of differentiation 163) and MACE (major adverse cardiovascular event; composite of stroke, myocardial infarction, or death).

Over a follow-up period of 2 years, individuals with high plasma concentrations of IL-6 based on the median value within our patient sample had a lower freedom from a MACE (HR 1.269 (95% CI 1.122–1.639), *p* = 0.042) (Figure 1). Similarly, individuals with high levels of CD163 based on the median value within our patient sample had lower freedom from a 2-year MACE (HR 1.413 (95% CI 1.022–1.954), *p* = 0.036) (Figure 2). Table 5 and Table 6 provide the population at risk at 12 and 24 months of follow-up for comparison of a 2-year MACE in patients with low vs. high plasma levels of IL-6 and CD163, respectively.

## 4. Discussion

### 4.1. Summary of Findings

Herein, we identified IL-6 and CD163 as CAS-specific biomarkers and demonstrated that these inflammatory proteins have good prognostic value in patients with CAS. These biomarkers can be used for relatively long-term prognosis with 2 years of follow-up. Several key findings emerged from our analysis. Firstly, 29 inflammatory proteins were analyzed, and we discovered two to be significantly elevated in individuals with CAS with a 2-year MACE compared to those without a 2-year MACE (IL-6 and CD163), as demonstrated in Section 3.2 of the results. Second, we demonstrated significant correlations between every one unit elevation in these two proteins and a 2-year MACE after adjusting for baseline characteristics, as demonstrated in Section 3.3 of the results. Third, we stratified patients into low vs. high levels of IL-6 and CD163 based on the median plasma concentrations of these proteins in our cohort and found that individuals with high plasma levels of these proteins had a lower freedom from a MACE over 2 years, as demonstrated in Section 3.4 of the results. This demonstrates the clinical relevance of our model in helping clinicians understand the future trajectory of their CAS patients in terms of the risk of adverse events to guide personalized decision-making regarding further evaluation, monitoring, and treatment.

### 4.2. Comparison to the Existing Literature

Previous papers have assessed the associations between IL-6/CD163 and CAS. Puz and colleagues (2013) showed that individuals with CAS had higher plasma concentrations of IL-6 [37]. Similarly, Bountouris et al. (2009) demonstrated that IL-6 levels were elevated in individuals with symptomatic vs. asymptomatic CAS [38]. Zhang and colleagues (2018) showed that IL-6 was associated with cardiovascular disease risk with co-association with hypertension and hypercholesterolemia in a pooled analysis of over 200,000 patients [39]. Others have shown a correlation between IL-6 and carotid plaque instability mediated by higher levels of local inflammation [40]. We similarly showed a correlation between IL-6 and CAS outcomes, with higher plasma IL-6 levels being a predictor of a 2-year MACE in patients with CAS. With regards to CD163, Bengtsson and colleagues (2020) showed that CD163+ macrophages were correlated with vulnerable carotid plaques [8]. Specifically, the authors analyzed 200 human carotid plaques and showed that CD163 expression was elevated in plaques from symptomatic individuals compared to asymptomatic patients [8]. Durda and colleagues (2022) showed an association between circulating soluble CD163 and cardiovascular events and mortality using a genome-wide association study [41]. David et al. (2020) demonstrated that CD163 was a biomarker for accelerated atherosclerosis, specifically progressive carotid plaque, in patients with systemic lupus erythematosus [42]. Elsewhere, Otsuka et al. (2017) showed that individuals with symptomatic CAS had an increased expression of CD163+ macrophages and adding anti-inflammatory drugs to reduce macrophage lipoprotein-associated phospholipase A2 expression may reduce inflammation and apoptosis and therefore limit the expansion of the necrotic core and progression of the lesion [43]. We took these findings a step further and demonstrated a significant association between plasma CD163 levels and adverse clinical events of a 2-year MACE in patients with CAS. Overall, our work contributes to the current literature by demonstrating the prognostic value of IL-6 and CD163 in predicting long-term adverse outcomes in patients with CAS. 

### 4.3. Explanation of Study Findings

IL-6 belongs to the family of pro-inflammatory cytokines and induces the expression of various proteins that contribute to inflammation [44]. It also facilitates the proliferation/differentiation of various human cell types [44,45]. The signaling of IL-6 is mediated by the transmembrane IL-6 receptor, soluble forms of IL-6R, and gp130 [44,46]. These pathways contribute to IL-6′s pleiotropic functions, including the synthesis of acute phase proteins (e.g., C-reactive protein), induction of T cell growth and differentiation, and upregulation of thymocyte proliferation, among other functions [44,47]. Importantly, IL-6 has been demonstrated to induce the endothelial expression of adhesion molecules and the production of monocyte chemoattractant protein-1, which is a chemokine that facilitates the recruitment of monocytes [48]. This is particularly important with regard to CAS as endothelial dysfunction and chronic inflammation are important contributors to atherosclerosis in the carotid arteries [9]. Therefore, this may explain the potential mechanism by which higher circulating levels of IL-6 may contribute to more vulnerable carotid plaques and therefore predict adverse events in patients with CAS [9]. Similarly, CD163 is a marker specific to monocytes and macrophages, and they are primarily expressed in cells that possess strong anti-inflammatory potential [49]. CD163 expression is induced by anti-inflammatory mediators including glucocorticoids and IL-10, while CD163 is inhibited by pro-inflammatory molecules including interferon-gamma [49]. Importantly, mononuclear phagocytes that express CD163 may facilitate the downregulation of inflammation [49]. The upregulation of CD163 is an important change characteristic of macrophages switching to alternatively activated phenotypes in the inflammation response [12]. Therefore, CD163 upregulation in macrophages is characteristic of tissues’ response to inflammation [12]. This is particularly relevant to CAS because macrophages are abundant in atherosclerotic plaques [8]. Macrophages that express CD163 are associated with intraplaque hemorrhage and demonstrate a proatherogenic role [8]. These findings may explain why higher levels of CD163 in plasma may contribute to carotid plaque progression and vulnerability and therefore predict adverse outcomes in patients with CAS [8].

### 4.4. Implications

This study offers practical implications for guiding personalized clinical decision-making across various scenarios. Firstly, our tool can be employed to assess patients with asymptomatic CAS, which is particularly valuable in family practice settings. General practitioners can integrate the measurement of IL-6 and CD163 into their clinical assessments to gauge an individual’s risk of CAS-related adverse outcomes. Individuals screening positive for being at a high risk of poor outcomes may be referred for further vascular evaluation and management by a specialist such as a vascular surgeon or vascular medicine specialist [50]. On the other hand, patients categorized as low risk may continue to receive care from their family physician, focusing on risk factor optimization through management options including statins, acetylsalicylic acid, and lifestyle changes [51]. Once a referral has been made, vascular specialists can assess the measured plasma levels of IL-6 and CD163 as indicators of the patient’s risk of potential adverse outcomes and use this information in addition to identify individuals at an elevated risk of a MACE who could benefit from (1) additional imaging to delineate vascular anatomy and severity of disease [52], (2) low-dose rivaroxaban [53], or (3) carotid endarterectomy or stenting in patients at the highest risk [54]. Our study may enhance care for patients with CAS in both generalist and specialist settings. It facilitates efficient personalized CAS risk-stratification and prompt identification of patients at high risk for adverse events. This, in turn, can reduce unnecessary specialist referrals, lead to improved CAS outcomes, and concurrently lower healthcare costs.

### 4.5. Limitations

This study has several limitations. Firstly, recruitment occurred at a single center, which may affect the generalizability of the results. Secondly, a longer follow-up may help strengthen our understanding of the long-term prognostic value of IL-6 and CD163 in CAS. Thirdly, this study excluded individuals with specific conditions including acute coronary syndrome, elevated troponin, or inflammatory disorders requiring biological anti-inflammatory medications within the previous 3 months to reduce potential heterogeneity in disease processes and outcomes. Therefore, our results only apply to select patients with CAS. Further investigation is required to validate the potential of CD163 and IL-6 as prognostic biomarkers in individuals with CAS.

## 5. Conclusions

Herein, we demonstrated that inflammatory biomarkers IL-6 and CD163 have prognostic value in CAS. Higher plasma IL-6 and CD163 levels are associated independently with a 2-year MACE in individuals with CAS. The measurement of circulating levels of IL-6 and CD163 may help identify individuals with CAS who are at an elevated risk of developing adverse outcomes. These patients could benefit from further evaluation by vascular specialists, aggressive management, and close follow-up. Additionally, our findings underscore the need for translational studies assessing the biological relationships between IL-6/CD163 and CAS development, which may provide greater insight into the underlying pathogenesis of CAS and inform personalized diagnostic and therapeutic strategies. This study has the potential for direct clinical impact by improving outcomes for patients with CAS. Further studies with more patients and a longer prospective follow-up are necessary to confirm these findings.

## Figures and Tables

**Table 1:** Baseline characteristics of individuals with vs. without carotid artery stenosis.

	No Carotid Artery Stenosis (n = 46)	Carotid Artery Stenosis (n = 290)	*p*
Age, mean (SD)	63.76 (13.38)	71.60 (8.55)	<0.001
Female sex	19 (42%)	110 (38%)	0.78
Male sex	27 (58%)	180 (62%)	0.78
Hypertension	25 (55%)	226 (78%)	<0.001
Dyslipidemia	23 (51%)	238 (82%)	<0.001
Diabetes	10 (22%)	90 (31%)	0.03
Current smoking	18 (40%)	157 (54%)	0.003
Congestive heart failure	3 (7%)	12 (4%)	0.16
Coronary artery disease	12 (27%)	107 (37%)	0.03
Previous stroke	10 (22%)	99 (34%)	0.005

Values reported as N (%) unless otherwise indicated. Abbreviation: SD (standard deviation).

**Table 2:** Plasma protein concentrations in individuals with carotid artery stenosis who developed vs. did not develop 2-year major adverse cardiovascular events.

	Did Not Develop 2-Year MACE	Developed 2-Year MACE	*p*-Value
IL-6	3.36 ± 4.04	5.07 ± 4.66	0.030
CD163	159.673 ± 175.669	233.825 ± 230.306	0.033
Reg3A	22.676 ± 20.778	31.098 ± 42.075	0.061
Resistin	5.807 ± 5.640	7.611 ± 6.703	0.101
MMP7	4.555 ± 2.773	3.788 ± 1.926	0.140
CEACAM-1/CD66a	16.352 ± 15.450	20.108 ± 16.919	0.209
TNFa	4.47 ± 2.96	5.17 ± 2.96	0.218
MMP8	234.433 ± 385.299	161.237 ± 262.682	0.310
BMP4	14.79 ± 7.15	16.16 ± 6.00	0.312
Progranulin/PGRN	22.855 ± 31.625	27.031 ± 23.704	0.482
CCL15/MIP-1 delta	2.180 ± 3.334	2.566 ± 2.560	0.539
HTRA2/Omi	0.977 ± 1.203	1.113 ± 1.073	0.551
IGFBP1	16.221 ± 31.167	19.538 ± 23.972	0.578
Serpin A12	0.703 ± 4.380	0.268 ± 0.871	0.588
MMP10	0.709 ± 0.610	0.770 ± 0.468	0.600
IFNy	12.747 ± 18.035	10.974 ± 16.303	0.605
CXCL9/MIG	4.360 ± 6.796	3.709 ± 5.287	0.611
Osteoactivin/GPNMB	16.836 ± 12.975	17.858 ± 8.487	0.673
CXCL16	8.470 ± 10.543	7.726 ± 9.815	0.711
CCL17/TARC	0.372 ± 1.262	0.287 ± 0.318	0.712
CCL1/TCA-3	24.18 ± 362.00	2.98 ± 2.16	0.749
CCL2/MCP1	2.598 ± 36.999	0.634 ± 1.085	0.772
BMP10	0.115 ± 0.108	0.109 ± 0.068	0.799
CCL4/MIP1b	6.769 ± 13.668	7.409 ± 13.170	0.806
TFPI	19.940 ± 10.307	19.557 ± 10.822	0.846
CCL11/Eotaxin	0.100 ± 0.203	0.105 ± 0.063	0.893
BMP7	0.134 ± 0.520	0.123 ± 0.072	0.911
Serpin B3/SCCA1	0.345 ± 0.701	0.354 ± 0.528	0.947
CCL3/MIP1a	1.353 ± 2.182	1.376 ± 2.354	0.957

Protein concentrations reported in mean ± SD (pg/mL). Abbreviations: MACE (major adverse cardiovascular event), MMP-7 (matrix metalloproteinase-7), MMP-10 (matrix metalloproteinase-10), IL-6 (interleukin-6), CCL-2/MCP-1 (monocyte chemoattractant protein), TFPI (tissue factor pathway inhibitor), CCL17/TARC (chemokine (C-C motif) ligand 17/thymus and activation regulated chemokine), Reg3A (regenerating family member 3 alpha), MIP-1a (macrophage inflammatory protein-1 alpha), IGFBP-1 (insulin-like growth factor-binding protein-1), BMP-4 (bone morphogenetic protein 4), CD163 (cluster of differentiation 163), CXCL16 (chemokine (C-X-C motif) ligand 16), TNFa (tumor necrosis factor alpha), CEACAM1/CD66a (carcinoembryonic antigen-related cell adhesion molecule 1), GPNMB (glycoprotein (transmembrane) NMB), IFNy (interferon gamma), and SCCA1 (squamous cell carcinoma antigen 1).

**Table 3:** Major adverse cardiovascular events at 1 year and 2 years of follow-up for patients with and without carotid artery stenosis.

	No Carotid Artery Stenosis (n = 46)	Carotid Artery Stenosis (n = 290)
MACE at 1 year	0 (0)	23 (7.93%)
MACE at 2 years	4 (8.70%)	30 (10.34%)

Values reported as N (%). MACE: major adverse cardiovascular event.

**Table 4:** Hazard ratios for 2-year major adverse cardiovascular events per every one unit increase in plasma IL-6 and CD163 concentrations.

	Adjusted HR (95% CI) *	*p*
IL-6	1.269 (1.122–1.639)	0.042
CD163	1.413 (1.022–1.954)	0.036

* Adjusted for sex, age, dyslipidemia, hypertension, diabetes, current smoking, congestive heart failure, coronary artery disease, and previous stroke. Abbreviations: CD163 (cluster of differentiation 163), IL-6 (interleukin-6), CI (confidence interval), and HR (hazard ratio).

**Table 5:** Population at risk at 12 and 24 months of follow-up for comparison of 2-year major adverse cardiovascular events in patients with low vs. high plasma levels of interleukin-6.

	Low IL-6 (N = 168)	High IL-6 (N = 168)
12 months	166 (99%)	147 (87%)
24 months	163 (97%)	139 (83%)

**Table 6:** Population at risk at 12 and 24 months of follow-up for comparison of 2-year major adverse cardiovascular events in patients with low vs. high plasma levels of cluster of differentiation 163.

	Low CD163 (N = 138)	High CD163 (N = 138)
12 months	135 (97%)	118 (86%)
24 months	134 (95%)	108 (78%)

## Data Availability

The original contributions presented in this study are included in the article; further inquiries can be directed to the corresponding author.

## References

[1] Qaja E., Tadi P., Theetha Kariyanna P. (2024). Carotid Artery Stenosis. StatPearls.

[2] Donkor E.S. (2018). Stroke in the 21st Century: A Snapshot of the Burden, Epidemiology, and Quality of Life. Stroke Res. Treat..

[3] Shimoda S., Kitamura A., Imano H., Cui R., Muraki I., Yamagishi K., Umesawa M., Sankai T., Hayama-Terada M., Kubota Y. (2020). Associations of Carotid Intima-Media Thickness and Plaque Heterogeneity with the Risks of Stroke Subtypes and Coronary Artery Disease in the Japanese General Population: The Circulatory Risk in Communities Study. J. Am. Heart Assoc..

[4] Ismail A., Ravipati S., Gonzalez-Hernandez D., Mahmood H., Imran A., Munoz E.J., Naeem S., Abdin Z.U., Siddiqui H.F. (2023). Carotid Artery Stenosis: A Look Into the Diagnostic and Management Strategies, and Related Complications. Cureus.

[5] Johri A.M., Nambi V., Naqvi T.Z., Feinstein S.B., Kim E.S.H., Park M.M., Becher H., Sillesen H. (2020). Recommendations for the Assessment of Carotid Arterial Plaque by Ultrasound for the Characterization of Atherosclerosis and Evaluation of Cardiovascular Risk: From the American Society of Echocardiography. J. Am. Soc. Echocardiogr..

[6] Brinjikji W., Huston J., Rabinstein A.A., Kim G.-M., Lerman A., Lanzino G. (2016). Contemporary carotid imaging: From degree of stenosis to plaque vulnerability. J. Neurosurg..

[7] Migdalski A., Jawien A. (2021). New insight into biology, molecular diagnostics and treatment options of unstable carotid atherosclerotic plaque: A narrative review. Ann. Transl. Med..

[8] Bengtsson E., Hultman K., Edsfeldt A., Persson A., Nitulescu M., Nilsson J., Gonçalves I., Björkbacka H. (2020). CD163+ macrophages are associated with a vulnerable plaque phenotype in human carotid plaques. Sci. Rep..

[9] Kamtchum-Tatuene J., Saba L., Heldner M.R., Poorthuis M.H.F., de Borst G.J., Rundek T., Kakkos S.K., Chaturvedi S., Topakian R., Polak J.F. (2022). Interleukin-6 Predicts Carotid Plaque Severity, Vulnerability, and Progression. Circ. Res..

[10] Tyrrell D.J., Goldstein D.R. (2021). Ageing and atherosclerosis: Vascular intrinsic and extrinsic factors and potential role of IL-6. Nat. Rev. Cardiol..

[11] Gabay C. (2006). Interleukin-6 and chronic inflammation. Arthritis Res. Ther..

[12] Etzerodt A., Moestrup S.K. (2013). CD163 and inflammation: Biological, diagnostic, and therapeutic aspects. Antioxid. Redox Signal.

[13] Sakamoto A., Kawakami R., Mori M., Guo L., Paek K.H., Mosquera J.V., Cornelissen A., Ghosh S.K.B., Kawai K., Konishi T. (2023). CD163+ macrophages restrain vascular calcification, promoting the development of high-risk plaque. JCI Insight.

[14] Ceasovschih A., Sorodoc V., Onofrei (Aursulesei) V., Tesloianu D., Tuchilus C., Anisie E., Petris A., Statescu C., Jaba E., Stoica A. (2020). Biomarker Utility for Peripheral Artery Disease Diagnosis in Real Clinical Practice: A Prospective Study. Diagnostics.

[15] Kremers B., Wübbeke L., Mees B., Ten Cate H., Spronk H., Ten Cate-Hoek A. (2020). Plasma Biomarkers to Predict Cardiovascular Outcome in Patients with Peripheral Artery Disease: A Systematic Review and Meta-Analysis. Arterioscler. Thromb. Vasc. Biol..

[16] Zakynthinos E., Pappa N. (2009). Inflammatory biomarkers in coronary artery disease. J. Cardiol..

[17] Gyanwali B., Lai M.K.P., Lui B., Liew O.W., Venketasubramanian N., Richards A.M., Chen C., Hilal S. (2021). Blood-Based Cardiac Biomarkers and the Risk of Cognitive Decline, Cerebrovascular Disease, and Clinical Events. Stroke.

[18] Khan H., Shaikh F., Syed M.H., Mamdani M., Saposnik G., Qadura M. (2023). Current Biomarkers for Carotid Artery Stenosis: A Comprehensive Review of the Literature. Metabolites.

[19] Antonopoulos A.S., Angelopoulos A., Papanikolaou P., Simantiris S., Oikonomou E.K., Vamvakaris K., Koumpoura A., Farmaki M., Trivella M., Vlachopoulos C. (2022). Biomarkers of Vascular Inflammation for Cardiovascular Risk Prognostication: A Meta-Analysis. JACC Cardiovasc. Imaging.

[20] Patoulias D., Stavropoulos K., Imprialos K., Athyros V., Grassos H., Doumas M., Faselis C. (2021). Inflammatory Markers in Cardiovascular Disease; Lessons Learned and Future Perspectives. Curr. Vasc. Pharmacol..

[21] Li H., Sun K., Zhao R., Hu J., Hao Z., Wang F., Lu Y., Liu F., Zhang Y. (2018). Inflammatory biomarkers of coronary heart disease. Front. Biosci. (Schol. Ed.).

[22] Mohebi R., McCarthy C.P., Gaggin H.K., van Kimmenade R.R.J., Januzzi J.L. (2022). Inflammatory biomarkers and risk of cardiovascular events in patients undergoing coronary angiography. Am. Heart J..

[23] Liu Y., Guan S., Xu H., Zhang N., Huang M., Liu Z. (2023). Inflammation biomarkers are associated with the incidence of cardiovascular disease: A meta-analysis. Front. Cardiovasc. Med..

[24] (2013). World Medical Association World Medical Association Declaration of Helsinki: Ethical principles for medical research involving human subjects. JAMA.

[25] Collins G.S., Reitsma J.B., Altman D.G., Moons K.G.M. (2015). Transparent Reporting of a multivariable prediction model for Individual Prognosis or Diagnosis (TRIPOD): The TRIPOD statement. Ann. Intern. Med..

[26] Chang Y.-J., Golby A.J., Albers G.W. (1995). Detection of Carotid Stenosis. Stroke.

[27] AbuRahma A.F., Avgerinos E.D., Chang R.W., Darling R.C., Duncan A.A., Forbes T.L., Malas M.B., Murad M.H., Perler B.A., Powell R.J. (2022). Society for Vascular Surgery clinical practice guidelines for management of extracranial cerebrovascular disease. J. Vasc. Surg..

[28] Ricotta J.J., AbuRahma A., Ascher E., Eskandari M., Faries P., Lal B.K. (2011). Updated Society for Vascular Surgery guidelines for management of extracranial carotid disease. J. Vasc. Surg..

[29] Grundy S.M., Stone N.J., Bailey A.L., Beam C., Birtcher K.K., Blumenthal R.S., Braun L.T., De Ferranti S., Faiella-Tommasino J., Forman D.E. (2019). 2018 AHA/ACC/AACVPR/AAPA/ABC/ACPM/ADA/AGS/APhA/ASPC/NLA/PCNA Guideline on the Management of Blood Cholesterol. J. Am. Coll. Cardiol..

[30] Whelton P.K., Carey R.M., Aronow W.S., Casey D.E., Collins K.J., Dennison H.C., DePalma S.M., Gidding S., Jamerson K.A., Jones D.W. (2018). 2017 ACC/AHA/AAPA/ABC/ACPM/AGS/APhA/ASH/ASPC/NMA/PCNA Guideline for the Prevention, Detection, Evaluation, and Management of High Blood Pressure in Adults. J. Am. Coll. Cardiol..

[31] Luminex Assays, Multiplex Immunoassays. Bio-Techne n.d. https://www.bio-techne.com/.

[32] Luminex Assays—CA, n.d. https://www.thermofisher.com/ca/en/home/life-science/antibodies/immunoassays/procartaplex-assays-luminex.html.

[33] MAGPIX® System|xMAP Instrument|Luminex Corporation, n.d. https://www.luminexcorp.com/magpix-system/.

[34] xPONENT® Software for xMAP® Instruments. Luminex Corporation n.d. https://www.luminexcorp.com/xponent/.

[35] Riley R.D., Ensor J., Snell K.I.E., Harrell F.E., Martin G.P., Reitsma J.B., Moons K.G.M., Collins G., van Smeden M. (2020). Calculating the sample size required for developing a clinical prediction model. BMJ.

[36] (2021). SPSS Software. https://www.ibm.com/analytics/spss-statistics-software.

[37] Puz P., Lasek-Bal A., Ziaja D., Kazibutowska Z., Ziaja K. (2013). Inflammatory markers in patients with internal carotid artery stenosis. Arch. Med. Sci..

[38] Bountouris I., Paraskevas K.I., Koutouzis M., Tzavara V., Nikolaou N., Nomikos A., Barbatis C., Andrikopoulos V., Mikhailidis D.P., Andrikopoulou M. (2009). Serum leptin levels in patients undergoing carotid endarterectomy: A pilot study. Angiology.

[39] Zhang B., Li X.-L., Zhao C.-R., Pan C.-L., Zhang Z. (2018). Interleukin-6 as a Predictor of the Risk of Cardiovascular Disease: A Meta-Analysis of Prospective Epidemiological Studies. Immunol. Investig..

[40] Shindo A., Tanemura H., Yata K., Hamada K., Shibata M., Umeda Y., Asakura F., Toma N., Sakaida H., Fujisawa T. (2014). Inflammatory biomarkers in atherosclerosis: Pentraxin 3 can become a novel marker of plaque vulnerability. PLoS ONE.

[41] Durda P., Raffield L.M., Lange E.M., Olson N.C., Jenny N.S., Cushman M., Deichgraeber P., Grarup N., Jonsson A., Hansen T. (2022). Circulating Soluble CD163, Associations with Cardiovascular Outcomes and Mortality, and Identification of Genetic Variants in Older Individuals: The Cardiovascular Health Study. J. Am. Heart Assoc..

[42] David C., Divard G., Abbas R., Escoubet B., Chezel J., Chauveheid M.P., Rouzaud D., Boutten A., Papo T., Dehoux M. (2020). Soluble CD163 is a biomarker for accelerated atherosclerosis in systemic lupus erythematosus patients at apparent low risk for cardiovascular disease. Scand. J. Rheumatol..

[43] Otsuka F., Zhao X., Trout H.H., Qiao Y., Wasserman B.A., Nakano M., Macphee C.H., Brandt M., Krug-Gourley S., Guo L. (2017). Community-based statins and advanced carotid plaque: Role of CD163 positive macrophages in lipoprotein-associated phospholipase A2 activity in atherosclerotic plaque. Atherosclerosis.

[44] Uciechowski P., Dempke W.C.M. (2020). Interleukin-6: A Masterplayer in the Cytokine Network. Oncology.

[45] Li B., Jones L.L., Geiger T.L. (2018). IL-6 Promotes T Cell Proliferation and Expansion under Inflammatory Conditions in Association with Low-Level RORγt Expression. J. Immunol..

[46] Mihara M., Hashizume M., Yoshida H., Suzuki M., Shiina M. (2012). IL-6/IL-6 receptor system and its role in physiological and pathological conditions. Clin. Sci..

[47] Hirano T. (2021). IL-6 in inflammation, autoimmunity and cancer. Int. Immunol..

[48] Romano M., Sironi M., Toniatti C., Polentarutti N., Fruscella P., Ghezzi P., Faggioni R., Luini W., van Hinsbergh V., Sozzani S. (1997). Role of IL-6 and its soluble receptor in induction of chemokines and leukocyte recruitment. Immunity.

[49] Kowal K., Silver R., Sławińska E., Bielecki M., Chyczewski L., Kowal-Bielecka O. (2011). CD163 and its role in inflammation. Folia Histochem. Cytobiol..

[50] Cameron S.J. (2015). Vascular Medicine. J. Am. Coll. Cardiol..

[51] Hackam D.G. (2021). Optimal Medical Management of Asymptomatic Carotid Stenosis. Stroke.

[52] U-King-Im J.M., Young V., Gillard J.H. (2009). Carotid-artery imaging in the diagnosis and management of patients at risk of stroke. Lancet Neurol..

[53] Perera K.S., Ng K.K.H., Nayar S., Catanese L., Dyal L., Sharma M., Connolly S.J., Yusuf S., Bosch J., Eikelboom J.W. (2020). Association Between Low-Dose Rivaroxaban With or Without Aspirin and Ischemic Stroke Subtypes: A Secondary Analysis of the COMPASS Trial. JAMA Neurol..

[54] Reiff T., Eckstein H.-H., Mansmann U., Jansen O., Fraedrich G., Mudra H., Böckler D., Böhm M., Debus E.S., Fiehler J. (2022). Carotid endarterectomy or stenting or best medical treatment alone for moderate-to-severe asymptomatic carotid artery stenosis: 5-year results of a multicentre, randomised controlled trial. Lancet Neurol..

